# Influence of Different Aggregation States on Volatile Organic Compounds Released by Dairy *Kluyveromyces marxianus* Strains

**DOI:** 10.3390/foods11182910

**Published:** 2022-09-19

**Authors:** Giorgia Perpetuini, Fabrizia Tittarelli, Carlo Perla, Rosanna Tofalo

**Affiliations:** 1 Faculty of Bioscience and Technology for Food, Agriculture and Environment, University of Teramo, Via R. Balzarini 1, 64100 Teramo, Italy; 2 Dalton Biotecnologie s.r.l., 65010 Spoltore, Italy

**Keywords:** *Kluyveromyves marxianus*, volatile organic compounds, biofilm, MATS, dairy products

## Abstract

*Kluyveromyces marxianus* has the ability to contribute to the aroma profile of foods and beverages since it is able to produce several volatile organic compounds (VOCs). In this study, 8 dairy *K. marxianus* strains, previously selected for their adhesion properties, were tested for VOCs production when grown in different conditions: planktonic, biofilm-detached, and MATS forming-cells. It was shown that biofilm-detached cells were mainly able to produce higher alcohols (64.57 mg/L), while esters were mainly produced by planktonic and MATS forming-cells (117.86 and 94.90 mg/L, respectively). Moreover, *K. marxianus* biofilm-detached cells were able to produce VOCs with flavor and odor impacts, such as ketons, phenols, and terpenes, which were not produced by planktonic cells. In addition, specific unique compounds were associated to the different conditions tested. Biofilm-detached cells were characterized by the production of 9 unique compounds, while planktonic and MATS forming-cells by 7 and 12, respectively. The obtained results should be exploited to modulate the volatilome of foods and beverages and improve the production of certain compounds at the industrial level. Further studies will be carried out to better understand the genetic mechanisms underlying the metabolic pathways activated under different conditions.

## 1. Introduction

The scientific interest for non-conventional yeast species is increasing since they can be exploited in food fermentations for their technological traits. Among the non-*Saccharomyces* yeasts, the species of the genus *Kluyveromyces* have a high potential for many industrial applications, especially for their ability to utilize low-cost substrates and high biomass production [1]. Particularly, *K. lactis* was the first species after *Saccharomyces cerevisiae* used as a model for the studies on lactose metabolism [2]. *Kluyveromyces marxianus*, a sister species of *K. lactis*, has gained particular research interest, due to its beneficial traits that render it exceptionally suitable for industrial applications [3]. These features include the ability to utilize a broad range of sugars including lactose and inulin, thermotolerance, secretion of lytic enzymes, the highest growth rate than other eukaryotes, production of bio-ingredients and aroma compounds, production of ethanol by fermentation, removal of lactose from food, and bioremediation [4]. In particular, this yeast species is reported to play a key role in cheese deacidification, release of autolytic compounds, and the development of flavor compounds during cheese ripening [3,5]. Moreover, thanks to its long and safe association with foods, it gained the GRAS (Generally Regarded As Safe) status in the USA and is on the European Food Safety Authority QPS (Qualified Presumption Of Safety) list. It has been reported that *K. marxianus* was the principal species in Pecorino di Farindola–an ewe’s milk cheese–and, Parmigiano Reggiano cheese, showing genotypic and phenotypic polymorphisms (growth kinetics in whey, production and degradation of organic acid, amino acid consumption, production of aroma metabolites) [6,7]. *K. marxianus* plays an important role in cheese ripening, being essential for the contribution to the final product quality, especially for what concerns the production of particular aroma compounds [8]. In fact, the principal interest of *K. marxianus* lies in its ability to produce volatile organic compounds (VOCs) such as fruit esters, carboxylic acids, ketones, furanes, and alcohols, e.g., 2-phenylethanol, which has a characteristic rose aroma, having a key role also in dairy product characterization [9].

In nature, microorganisms can alternate between two modes of growth: a unicellular life phase, in which the cells are free-swimming (planktonic); and a multicellular life phase, in which the cells are sessile and live in a biofilm which is microbial consortia, where cells stick to each other and often to a surface. Alternation between the two phases requires the transition from planktonic cells to sessile cells to initiate biofilm formation, and from sessile cells to detached cells to allow a return to the planktonic state. Sessile cells are embedded and protected within an extracellular matrix made up of extracellular polymeric substances produced by the same microorganisms involved [10]. The ability of yeasts to adhere and form biofilm on food matrices is essential for their growth and influences the final product quality [10]. In fact, microorganisms growing in biofilms show a different phenotype from their planktonic counterparts [10,11]. Recent studies have reported that biofilm-detached cells are characterized by phenotypes and properties different from those of their planktonic counterparts. For instance, in *Candida albicans*, the biofilm-detached cells better adhered to endothelial cells and caused increased damage compared with planktonic cells [12]. In *Pseudomonas aeruginosa*, the biofilm-detached cells showed a longer lag phase, suggesting that they were less able to return to the planktonic lifestyle. Moreover, biofilm-detached cells adhered more easily to surfaces than planktonic ones [13]. Recent studies performed on *Oenococcus oeni* revealed that planktonic and biofilm-detached cells showed a different volatilome [14,15]. Perpetuini et al. [16] highlighted that biofilm-detached cells of *Candida zemplinina* induced an increase in the glycerol and esters amounts, and a reduction in ethanol content in red wines. Some specific esters compounds such as methyl vanillate, ethyl isobutyrate, and ethyl isovalerate were produced only by sessile cells. Moreover, microorganisms can also form MAT structures (MATS), which can be considered an elaborate multicellular biofilm related to the sliding motility. According to Recht et al. [17], sliding motility is defined as a form of surface motility “produced by the expansive forces of the growing microbial population in combination with cell surface properties that favour reduced friction between the cells and the substrate”. MATS are formed on semisolid agar surfaces (0.3%) and not in liquid, and their formation is dependent upon the nature of the surface, the concentration of glucose, and the genetic background of the strain. As the microorganisms grow on the wet surface of a semisolid agar petri plate, they form MATS that spread over the agar. MATS are characterized by an interior region called the hub, which is distinguished by channels and wrinkles, and a smooth periphery called the rim [18]. 

At present, limited information is available regarding the physiological properties of *K. marxianus* biofilm-detached cells and whether they possess properties similar to those of planktonic or sessile cells. A recent study showed differences in dairy *K. marxianus* strains based on MATS’ formation and adhesion properties [19]. Therefore, the main goal of this study was to identify the metabolites that were differentially produced by biofilm-detached cells, or planktonic cells of *K. marxianus*, or when forming MATS. 

## 2. Materials and Methods

### 2.1. Strains’ Origin

Seven *K. marxianus* strains–M83, LM142, 1SC4, FM09, VG4, 6M2, M135–isolated from different dairy products were analyzed in this study [7,20,21]. The type strain *K. marxianus* CBS834^T^ was also included. The strains were routinely grown in YPD medium (yeast extract 1% *w*/*v*, peptone 2% *w*/*v*, glucose 2% *w*/*v*) overnight at 28 °C. Strains were stored at −80 °C in YPD broth supplemented with glycerol (20% *v*/*v* final concentration) or on YPD agar at 4 °C for short-term storage. All strains belong to the Culture Collection of the Microbial Biotechnology Laboratory (University of Teramo, Teramo, Italy).

### 2.2. Cells Obtainment in MATS, Planktonic, and in Biofilm-Detached States

**Figure 1:**
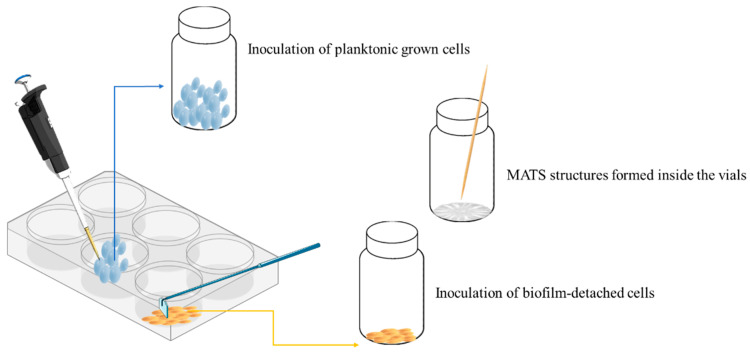
Obtainment of planktonic, biofilm-detached, and MATS forming-cells. Planktonic and sessile cells were obtained in 6-wells plates. Planktonic cells were removed pipetting up and down with PBS. Sessile cells were removed using a sterile cell scraper and inoculated in the vials as biofilm-detached cells. MATS were formed directly inside the vials.

Biofilms were formed inoculating cells in flat-bottom 6-well cell culture plates (Costar, Corning, NY, USA) containing 5 mL of YPD. Plates were incubated at 28 °C for 7 days in order to reach the stationary growth phase and to favor the formation of biofilms. The stationary phase was verified evaluating the OD_600nm_ with a Jenway 6305 UV–visible spectrophotometer. Planktonic and biofilm-detached cells were obtained as follows. Planktonic and loosely attached cells were removed by rinsing with phosphate-buffered saline (PBS; pH 7.4). Sessile cells were detached from the bottom of the well by being scraped off the surface of the well using a sterile cell scraper. Once removed from the bottom of the well, these cells were referred to as biofilm-detached cells. Both planktonic and biofilm-detached cells were serially diluted and plated on a YPD medium for the cell count. A final concentration of 10^6^ CFU/mL was inoculated in vials closed with a butyl/PTFE septum and a cap used for the Gas Chromatography/Mass Spectrometry (GC/MS) containing 5 mL of fresh YPD. MATS were developed directly inside the vials containing YPD medium supplemented with 0.3% (*w*/*v*) agar (Figure 1). In particular, yeast strains were inoculated in the center on the vials using a toothpick. Planktonic, biofilm-detached cells and MATS forming-cells were incubated at 28 °C for 7 days in order to detect also secondary metabolites. This time was necessary to allow the formation of the MATS. All analyses were performed in triplicate.

### 2.3. Volatile Organic Compounds Determination

Five milliliters of samples were placed in 10 mL glass vials with 1 g NaCl and 10 μL of 2-methyl-exanol (final concentration 0.1 M) were added as the internal standard. Both equilibration and adsorption phases were carried out by stirring for 30 min at 40 °C. 

The analysis of VOCs was performed by GC/MS using a GC-mass spectrometer Clarus SQ8S chromatography/mass (GC-MS) spectrometry (Perkin Elmer, Boston, MA, USA) as previously described [16,22]. The column used was a capillary GC column (30 m × 0.25 mm i.d, 0.25 μm film thickness) coated with polyethyleneglycol (film thickness 1.2 μm), as the stationary phase. A carboxen–polydimethylsiloxane-coated fiber (85 μm) was injected into the GC/MS, for VOCs identification for 15 min, and the following program was applied: 50 °C for 2 min; first ramp, 1 °C/min to 65 °C; second ramp, 10 °C/min to 150 °C (10 min hold); third ramp, 10 °C/min to 200 °C (1 min hold). The comparison of MS fragmentation patterns with those present in the National Institute for Standards and Technology database (NIST version 2005) was adopted to achieve a tentative identification. All determinations were performed in triplicate.

### 2.4. Statistical Analysis

Data were analyzed by means of the Prism 7.0 program (GraphPad Software Inc., La Jolla, CA, USA) and were expressed as mean value ± standard deviation. ANOVA was performed by means of the Prism 7.0 program. A level of *p* < 0.05 was considered statistically significant. A principal component analysis (PCA) based on the main volatile compounds released by strains was performed using XLStat 2014 software (Addinsoft, New York, NY, USA). Venn diagrams were generated to show the number of VOCs that are restricted to a specific lifestyle (planktonic, biofilm-detached, and MATS forming-cells), and the number of compounds that are present in two or more aggregation states. Venn diagrams were constructed using an open web tool provided by the Bioinformatics and Systems Biology of Gent (URL: http://bioinformatics.psb.ugent.be/webtools/Venn/ (accessed on 30 May 2022).

## 3. Results and Discussion

**Figure 2:**
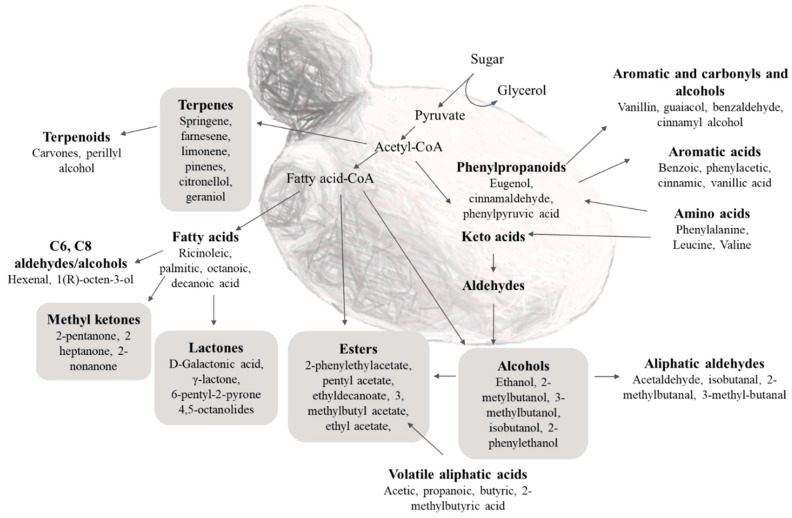
Main metabolic pathways in *K. marxianus*. Modified from Djordjević et al. [23], and Schrader [24].

Yeasts are known to release a wide array of VOCs with high chemical diversity, including esters, higher alcohols, organic acids, terpenoids, and ketones [23,24] (Figure 2). 

The majority of studies focused on their determination in planktonic cells. However, in nature, microorganisms mainly develop forming multicellular structures and several studies showed that planktonic, and biofilm-detached cells show a different metabolism [25,26,27]. This study investigated the volatilome of eight *K. marxianus* strains grown under different conditions: planktonic, biofilm-detached, and MATS forming-cells. 

### 3.1. Complexity of K. marxianus Volatilome under Different Conditions

**Figure 3:**
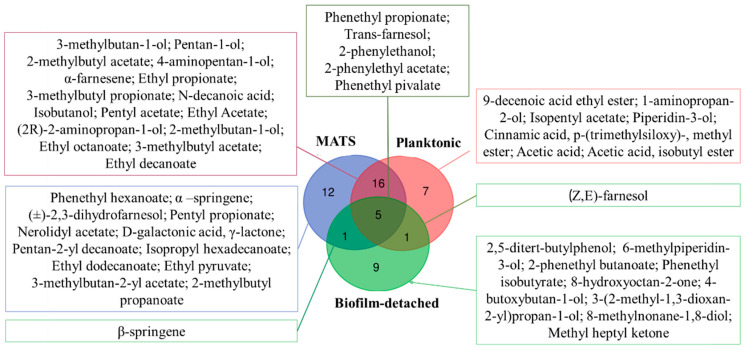
Venn diagram depicting the similarities and differences of VOCs produced in the different lifestyles.

The levels of VOCs produced by tested strains are shown in Table S1 and Figure 3. 

Significant differences were observed between the three different conditions in terms of the quantity and quality of VOCs produced (*p* < 0.05). Esters were mainly released by planktonic and MATS forming-cells (117.86 and 94.90 mg/L, respectively), while biofilm-detached cells mainly produced higher alcohols (64.57 mg/L). *K. marxianus* biofilm-detached cells were able to produce VOCs with high odor and sensory impact, such as ketones, phenols, and terpenes. However, biofilm-detached cells were not able to produce organic acids, and lactones. In this work, it was possible to find a core of five specific compounds (phenethyl propionate, phenethyl pivalate, trans-farnesol, 2-phenylethanol–2-PE, and 2-phenylethyl acetate–2-PEA) produced in all the conditions tested. Moreover, it was possible to find specific unique compounds present only in biofilm-detached cells, planktonic, or MATS forming-cells, suggesting that these compounds could have a quorum-sensing role in determining the aggregation state of the cells inducing phenotypic adaptations that include morphological changes, and biofilm formation [10].

In order to better highlight the differences among the aggregation states, Venn diagrams were generated. All the compounds produced under the different lifestyles were considered. In particular, biofilm-detached cells were characterized by the production of 9 unique compounds, while planktonic and MATS forming-cells by 7 and 12 compounds, respectively (Figure 2). Sixteen compounds were found in common between MATS forming-cells and planktonic cells, while MATS forming-cells and biofilm-detached cells and planktonic and biofilm-detached cells shared only one compound: β-springene, and (Z,E-farnesol, respectively (Figure 3). 

One of the main classes of VOCs produced were higher alcohols. They are synthesized from a carbon source or from the degradation of amino acids via the Ehrlich pathway, from branched-chain amino acids, leucine, valine, and isoleucine; aromatic amino acids, phenylalanine, tyrosine, and tryptophan; and the sulphur-containing amino acid methionine (Figure 2) [28]. Apart from 2-PE, the aroma contribution of the individual higher alcohols is not considered to be pleasant in fermented foods, particularly at high concentrations [29]. *K. marxianus* planktonic and MATS forming-cells mainly produced pentan-1-ol (20.28 and 33.15 mg/L, respectively), followed by 2-methylbutan-1-ol (9.26 and 12.22 mg/L, respectively), and 3-methylbutan-1-ol (8.97 and 11.30 mg/L, respectively). *K. marxianus* biofilm-detached cells produced the highest amount of 2-PE (61.81 mg/L), which was 7 times higher in comparison with the other states. In fact, planktonic and MATS forming-cells were able to produce only 2.43 and 4.96 mg/L of 2-PE, respectively. During fermentations, *K. marxianus* generally releases low levels of 2-PE [30]. This study highlighted that the living state of yeasts has a strong influence on the production of specific metabolites, and it could be possible to exploit the aggregation state to improve the production of certain VOCs including 2-PE. The acetate esters of higher alcohols, such as ethyl acetate (solvent-like aroma), 3-methylbutyl acetate (sweet, fruity, banana), ethyl octanoate (fruity, sweet, apricot, banana, pear, musty, pineapple, dairy, creamy, mushroom, cognac), and 2-PEA (floral, rose, sweet, honey, fruity, tropical, green) are important in enhancing fruity aromas [31,32]. The major acetate ester produced by *K. marxianus* planktonic and MATS forming-cells was 2-PEA (39.77 and 39.09 mg/L, respectively) followed by pentyl acetate (34.59 and 22.08 mg/L, respectively), ethyl decanoate (20.09 and 11.63 mg/L, respectively), ethyl acetate (13.48 and 8.80 mg/L, respectively), and 3-methylbutyl acetate (5.65 and 9.69 mg/L, respectively). Biofilm-detached cells produced lower levels of 2-PEA (15.39 mg/L). In this study, it was found that *K. marxianus* yeast biofilm-detached cells produced 2-phenethyl butanoate, and phenethyl isobutyrate which are usually found in low amounts and are sought after by the food industries for their sensory impact on foods [33]. Ketones and phenols were produced only by biofilm-detached cells. In particular, the detected compounds in *K. marxianus* biofilm-detached cells were methyl heptyl ketone (2-nonanone), and 8-hydroxyoctan-2-one. The ability of *K. marxianus* to produce ketones has been shown also by Risner et al. [34]. These authors found differences in ketones production by *K. marxianus* between acid and sweet whey distillates. These differences may be due to an increase in free fatty acid concentration of the whey. These free fatty acids are oxidized to produce β-ketoacids, which in turn are oxidized to produce alkan-2-ones; however, most of these methyl ketones were not found in acid whey distillates, suggesting another possible pathway for ketones formation [34]. The data obtained in this study suggested that in planktonic and MATS forming-cells, ketones could be oxygenated to esters; in fact, cells grown under these conditions showed higher concentrations of these compounds than biofilm-detached ones. A similar pathway has been well described in other yeasts such as *Candida maltosa* [35] and bacteria [36]. This reaction is found in organisms that utilizes ketones as a carbon source via a pathway involving ester formation followed by hydrolysis and metabolism of the released acid [34]. *K. marxianus* biofilm-detached cells produced terpenes such as farnesene and springene which are important odor compounds with floral notes [37]. The production of terpenes was lower in MATS forming-cells and planktonic cells, probably because β-glucosidase are mainly active under certain growth conditions. D-galactonic acid, γ-lactone was produced only by *K. marxianus* M83 MATS forming-cells. Probably, it is a strain specific trait since only a strain showed this ability. This evidence could be due to the genetic variability among strains. These differences between yeast strains could also lead to different flux through the pathway involved in lactones formation, with the potential for certain strains to produce D-galactonic acid, γ-lactone. The fact that it was produced only by MATS forming-cells might be related to a down-regulation of the pathways involved in lactones degradation. 

### 3.2. Klyveromyces Marxianus Strains’ Volatilome

**Figure 4:**
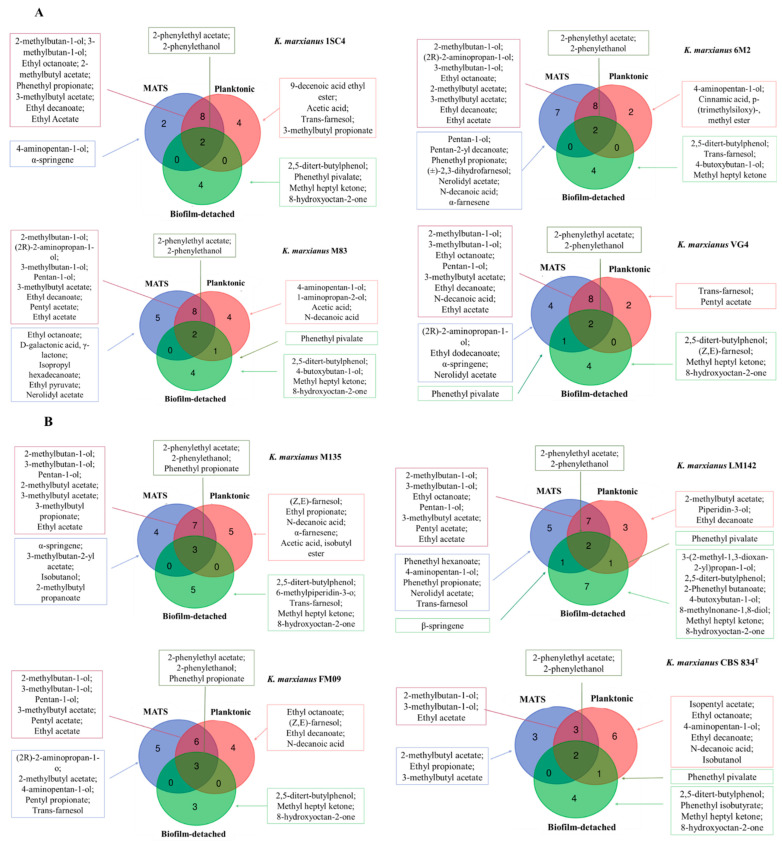
Similarities and differences of volatile compounds released by tested strains in different lifestyles shown by Venn diagram. Strains were divided into 2 groups on the basis of compounds shared by MATS forming-cells and planktonic cells. (**A**) MATS and planktonic cells sharing 8 compounds; (**B**) MATS and planktonic cells sharing less than 8 compounds.

A strain-specific behavior was observed in the three states (planktonic, biofilm-detached, and MATS forming-cells) tested. A strain characteristic core of compounds made up of two or three compounds was observed (Figure 4A,B). ISC4, 6M2, CBS834^T^, LM142, M83, and VG4 produced 2-PE, 2-PEA, while FM09, and M135 released 2-PE, 2-PEA, and phenethyl propionate in all conditions tested. 

The production of 2-PE and its acetate ester–2-PEA–has been described in several yeast species including *S. cerevisiae*, *Meyerozyma guilliermondii*, *Pichia fermentans*, and *K. marxianus* from L-phenylalanine; through the Ehrlich pathway; or by de novo synthesis from sugars, through the Shikimate pathway [38,39]. Obtained data revealed that 2-PE is mainly produced by biofilm-detached cells, suggesting that it could act as a quorum sensing molecule for biofilm formation as previously observed for *S. cerevisiae*. Chen and Fink [40] observed that 2-PE stimulated pseudomycelium formation and regulated cell density in *S. cerevisiae* inducing the expression of *FLO*11 gene, which encodes a flocculating protein, through a Tpk2p-dependent mechanism. 

In this study, 2-PEA production was mainly influenced by the strain and not by the growth condition (Figure 4A,B). In fact, some strains released the highest amount when growing as biofilm-detached cells (VG4), while others as planktonic cells (M135, 1SC4) or when forming MATS structures (6M2, CBS834^T^, FM09, LM142, M83). Moreno-García et al. [41] compared the proteome of a *S. cerevisiae* flor yeast growing under a biofilm formation condition and with those obtained under no biofilm formation condition. These authors observed that this compound showed a higher odor threshold in biofilm-detached cells. The controversial results may be related to a different metabolic strain dependent behavior of *K. marxianus* than *S. cerevisiae*, and also to the different culture conditions applied. 

FM09, and M135 strains showed a core of compounds made up of 2-PE, 2-PEA, and phenethyl propionate (Figure 4A,B). Phenethyl propionate (rose, floral, honey) is commonly not synthesized by *S. cerevisiae*. The production of this compound has been described in some non-*Saccharomyces* such as *L. thermotolerans*, *Torulaspora delbrueckii*, and *K. marxianus* [33,42]. In particular, Güneser et al. [33] evaluated the effects of alginate entrapment on fermentation metabolites of *K. marxianus* grown in agrowastes that served as the liquid culture media. These authors observed that phenethyl propionate was mainly produced when *K. marxianus* cells were entrapped. In agreement with this observation, also in this study, the highest production was observed in biofilm-detached cells suggesting the activation of specific metabolic pathways in this yeast once grown under this aggregation state. Beyond this core of compounds, it was possible to identify some compounds shared by planktonic and MATS forming-cells, MATS forming-cells and biofilm-detached cells, and biofilm-detached and planktonic cells. Obtained data revealed that MATS forming-cells were more similar to planktonic than to biofilm-detached ones. In fact, they share a number of compounds ranging from 3 (CBS834^T^) to 8 (1SC4, 6M2, M83, VG4). It could be due to the nature of MATS structures. In fact, they are complex multicellular structures composed of yeast cells. Unlike biofilm colonies, MATS are thin, and the cells are differentiated into two distinct populations: the first one forms a ‘rim’, a smooth MATS periphery composed of non-adherent cells; whereas the second one forms a ‘hub’ at the center of the MATS, composed of adherent cells [43]. Probably, under the conditions applied in this study, the first group of cells were more active and showed a metabolism similar to planktonic cells. Moreover, the fact that biofilm-detached cells and MATS forming-cells did not necessarily show a similar behavior is highlighted by other studies which showed that, *K. marxianus* strains able to adhere and to form biofilm on abiotic surfaces, are not capable of forming a MATS structure [44]. Figure 3 revealed the presence of some compounds associated to the different aggregation states which were specific for each strain. The different volatile patterns suggested the activation of metabolic pathways strictly associated to the aggregation state and specific for the different strains. This observation is in line with other studies which revealed a certain intrastrain variability in *K. marxianus*, indicating a heterogeneous contribution to VOCs production and the importance of strain selection considering also the aggregation state to modulate fermented foods’ flavor and aroma.

### 3.3. PCA Analysis

**Figure 5:**
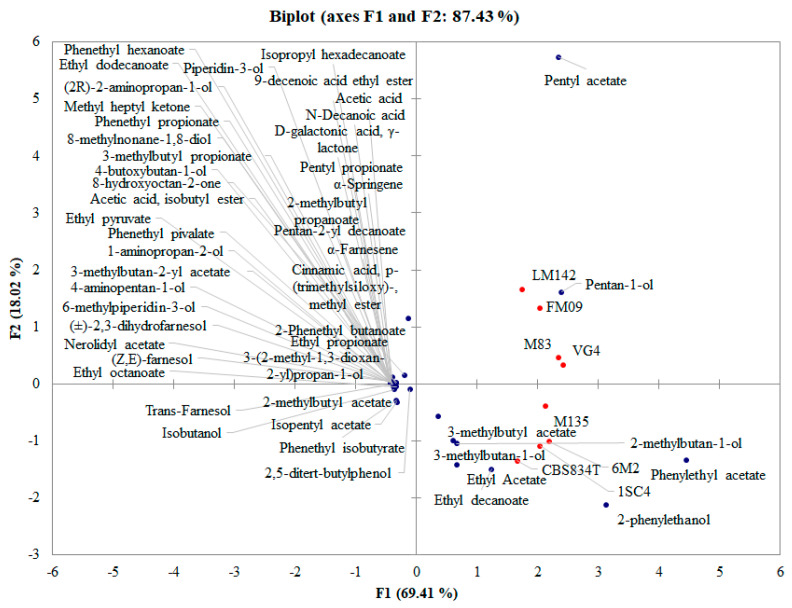
Principal component analysis (PCA) encompassing aroma compounds released by tested strains independently from the lifestyle.

A PCA was used to investigate the contribution of single strains (Figure 5) and yeast aggregation states (Figure 5) to VOCs release. To evaluate the contribution of the different strains, a PCA was performed considering all the compounds produced by the different strains, independently from the aggregation state. The PCA allowed 87.43% of the total variance, and F1 accounted for 69.41%, while F2 accounted for 18.02% (Figure 5). 

Based on the distribution of samples, two groups can be identified. The first is made up of LM142, FM09, M83, and VG4 strains, while the second one is by M135, CBS834^T^, 6M2, and M135 strains. Strains belonging to the first group were differentiated for pentyl acetate, while the others for 3-methylbutyl acetate, 2-methylbutan-1-ol, 3-methylbutan-1-ol, phenylethyl acetate, ethyl acetate, or 2-phenylethanol. Phenotypic heterogeneity of genetically identical cells can generate non-heritable variation in a population facilitating adaptation to adverse conditions in the wild [45]. Phenotypic variability can be considered a sort of evolving trait in a population facing a strong selective pressure of natural niches. This evidence could open new issues about evolution and phylogeny of this yeast. 

**Figure 6:**
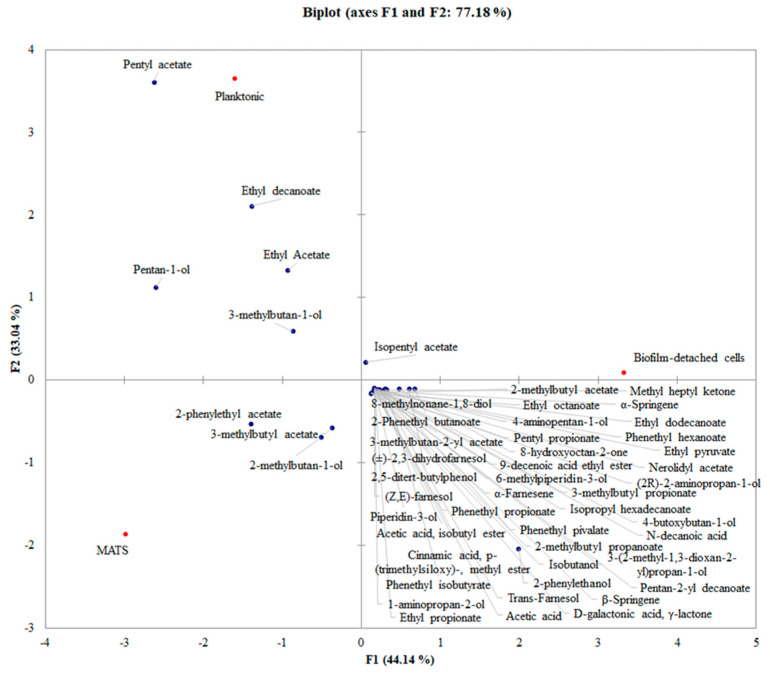
The biplot (score and loading) of the first two principal components encompassing aroma compounds released in the different lifestyle.

Figure 6 shows the impact of *K. marxianus* growth conditions on VOCs release. In this case, all the compounds produced by the tested strains under the different conditions were considered, independently from the strain. The PCA allowed 77.18% of the total variance to be explained by the first two PCs, and 44.14% was attributable to PC1 (Figure 6). The different lifestyles clustered separately, and MATS and planktonic states were both in the left part of the PCA graph. Planktonic cells were characterized by pentyl acetate, ethyl decanoate, ethyl acetate, 3-methylbutan-1-ol, and pentan 1-ol; MATS forming-cells by 2-phenylethyl acetate, 3-methylbutyl acetate, and 2-methylbutan-1-ol; while biofilm-detached cells were well differentiated for isopentyl acetate. Obtained data suggested that *K. marxianus* aggregation state exerted an effect in the definition of VOCs released, suggesting the presence of strain-specific metabolic pathways activated under specific conditions. 

## 4. Conclusions

Metabolic shifts are of crucial interest for the selection of candidate strains which could be used in green and sustainable biotechnological processes to shape the sensory traits of food products. The results obtained in this study allowed us to highlight the presence of *K. marxianus* strain-specific metabolic pathways associated to the aggregation state. In fact, biofilm-detached cells produced the highest concentration of higher alcohols, while planktonic and MATS forming-cells mainly released esters. Moreover, biofilm-detached cells were characterized by the production of 9 unique compounds, while planktonic and MATS forming-cells were characterized by 7 and 12, respectively. Some strains showed interesting traits. For instance, M135 and 1SC4 planktonic cells could be useful to accumulate 2-phenylethyl acetate in fermented foods, while M135, 6M2, and M183 biofilm-detached cells were characterized by the highest production of 2-phenylethanol. However, further studies based on genomic, transcriptomic, and enzyme activity assays on *K. marxianus* should be addressed to achieve this goal and gain useful information for innovative fermentation approaches in the near future.

## Data Availability

Data are contained within the article or Supplementary Material.

## Supplementary Materials

The following supporting information can be downloaded at: https://www.mdpi.com/article/10.3390/foods11182910/s1, Table S1: Main VOCs released by *K. marxianus* strains grown under different conditions.
