# Supplementary material for: Influence of Different Aggregation States on Volatile Organic Compounds Released by Dairy Kluyveromyces marxianus Strains
Source: Foods. 2022 Sep 19;11(18):2910. doi: 10.3390/foods11182910 (PMC9498923; doi:10.3390/foods11182910)
Supplement: Supplementary file 1 [file foods-11-02910-s001.zip › foods-1792728-supplementary.pdf]

Supplementary Table S1. Main VOCs released by *K. marxianus* strains grown under different conditions

Molecule	M135 MATS	M135 PLANK	M135 BIOFILM-DETACHED	Odor and Flavor
**Esters**				
2-methylbutyl acetate	0.13 ± 0.01	0.07 ± 0.01	N.d.	Sweet, fruity, banana, tropical, juicy
2-methylbutyl propanoate	0.10 ± 0.02	N.d.	N.d.	
3-methylbutan-2-yl acetate	0.89 ± 0.01	N.d.	N.d.	
3-methylbutyl acetate	1.49 ± 0.04	0.77 ± 0.02	N.d.	Sweet, fruity, banana
3-methylbutyl propionate	0.10 ± 0.01	0.05 ± 0.01	N.d.	Sweet, pineapple, tropical fruit, apple, banana, fresh green, melon
Acetic acid, isobutyl ester	N.d.	0.03 ± 0.01	N.d.	Sweet, fruity, banana, tropical, apple
Ethyl acetate	0.37 ± 0.01	1.42 ± 0.05	N.d.	Fruity, sweet, weedy, green, with a grape and cherry nuance
Ethyl propionate	N.d.	0.02 ± 0.01	N.d.	Sweet, fruity, juicy, grape, pineapple, fermented, winey, bubble gum, apple
Phenethyl propionate	0.01 ± 0.01	0.01 ± 0.01	0.11 ± 0.02	Floral, rose, fruity, honey, balsamic, sweet, rose, tropical
2-phenylethyl acetate	1.72 ± 0.03	10.58 ± 0.06	1.67 ± 0.05	Floral, rose, sweet, honey, fruity, tropical, rose, green
**Total Esters**	**4.84**	**12.97**	**1.78**	
**Alcohols**				
Trans-farnesol	N.d.	N.d.	0.69 ± 0.02	Muguet, floral, sweet, lily
(Z,E)-farnesol	N.d.	0.01 ± 0.01	N.d.	Delicate floral
2-methylbutan-1-ol	1.86 ± 0.05	0.96 ± 0.02	N.d.	Roasted, winey, fruity, alcoholic
2-phenylethanol	1.45 ± 0.06	0.16 ± 0.02	6.47 ± 0.09	Floral, rose, sweet, bready
3-methylbutan-1-ol	1.74 ± 0.05	0.90 ± 0.01	N.d.	Alcoholic, pungent, cognac, fruity, banana, molasses, fermented,
6-methylpiperidin-3-ol	N.d.	N.d.	0.58 ± 0.02	
Isobutanol	0.66 ± 0.02	N.d.	N.d.	Ethereal, winey, fusel, whiskey
Pentan-1-ol	9.21 ± 0.06	4.74 ± 0.06	N.d.	Fusel, oily, sweet, balsamic, pungent, fermented, bready, cereal, fruity
**Total Alcohols**	**14.93**	**6.78**	**7.75**	
**Ketons**				
Methyl heptyl ketone	N.d.	N.d.	0.31 ± 0.02	Fruity, sweet, cheesy, herbal, coconut, fresh, dairy, buttery
8-hydroxyoctan-2-one	N.d.	N.d.	1.13 ± 0.01	
**Total Ketons**	**N.d.**	**N.d.**	**1.45**	
**Organic acids**				
N-decanoic acid	N.d.	0.01 ± 0.01	N.d.	Rancid, sour, fatty, citrus, creamy, buttery, fruity, dairy
**Total Organic acids**	**N.d.**	**0,01**	**0**	
**Phenols**				
2,5-ditert-butylphenol	N.d.	N.d.	1.56 ± 0.02	
**Total Phenols**	**N.d.**	**N.d.**	**1.56**	
**Terpenes**				
α-springene	0.04 ± 0.01	N.d.	N.d.	
α-farnesene	N.d.	0.02 ± 0.01	N.d.	
**Total Terpenes**	**0.04**	**0.02**	**N.d.**	
**Molecule**	**6M2 MATS**	**6M2 PLANK**	**6M2 BIOFILM-DETACHED**	**Odor and Flavor**
**Esters**				
2-methylbutyl acetate	0.10 ± 0.01	0.16 ± 0.02	N.d.	Sweet, fruity, banana, tropical, juicy
3-methylbutyl acetate	1.09 ± 0.02	1.84 ± 0.02	N.d.	Sweet, fruity, banana
Cinnamic acid, p-(trimethylsiloxy)-, methyl ester	N.d.	0.02 ± 0.01	N.d.	
Ethyl acetate	0.49 ± 0.02	2.22 ± 0.02	N.d.	Fruity, sweet, weedy, green, with a grape and cherry nuance
Ethyl decanoate	3.09 ± 0.06	5.21 ± 0.07	N.d.	Sweet, waxy, fruity, apple, grape, oily, brandy
Ethyl octanoate	0.18 ± 0.02	0.31 ± 0.01	N.d.	Fruity, sweet, apricot, banana, pear, musty, pineapple, dairy, creamy
Nerolidyl acetate	0.03 ± 0.01	N.d.	N.d.	Fresh, sweet, citrus, freesia, woody

Pentan-2-yl decanoate	0.04 ± 0.01	N.d.	N.d.	
Phenethyl propionate	0.01 ± 0.01	N.d.	N.d.	Floral, rose, fruity, honey, balsamic, sweet, rose, tropical
2-phenylethyl acetate	4.99 ± 0.06	4.78 ± 0.07	0.62 ± 0.04	Floral, rose, sweet, honey, fruity, tropical, green
**Total Esters**	**10.04**	**14.58**	**0.628**	
**Alcohols**				
(±)-2,3-dihydrofarnesol	0.07 ± 0.01	N.d.	N.d.	Floral, green, fruity
(2R)-2-aminopropan-1-ol	0.06 ± 0.02	0.01 ± 0.01	N.d.	
2-methylbutan-1-ol	1.36 ± 0.05	2.29 ± 0.03	N.d.	Roasted, winey, fruity, alcoholic
2-phenylethanol	0.28 ± 0.04	0.53 ± 0.01	11.28 ± 0.09	Floral, rose, sweet, bready
3-methylbutan-1-ol	1.27 ± 0.05	2.15 ± 0.05	N.d.	Alcoholic, pungent, cognac, fruity, banana, molasses, fermented
4-aminopentan-1-ol	N.d.	0.04 ± 0.01	N.d.	
4-butoxybutan-1-ol	N.d.	N.d.	0.29 ± 0.02	
Pentan-1-ol	6.74 ± 0.07	N.d.	N.d.	Fusel, oily, sweet, balsamic, pungent, fermented, bready, cereal, fruity
Trans-farnesol	N.d.	N.d.	0.21 ± 0.01	Muguet, floral, sweet, lily
**Total Alcohols**	**9.80**	**5.02**	**11.78**	
**Ketons**				
Methyl heptyl ketone	N.d.	N.d.	0.23 ± 0.02	Fruity, sweet, cheesy, herbal, coconut, fresh, dairy, buttery
**Total Ketons**	**N.d.**	**N.d.**	**0.23** ± 0.01	
**Organic acids**				
N-decanoic acid	0.02 ± 0.01	N.d.	N.d.	Rancid, sour, fatty, citrus, creamy, buttery, fruity, dairy
**Total Organic acids**	**0.02**	**N.d.**	**N.d.**	
**Phenols**				
2,5-ditert-butylphenol	N.d.	N.d.	0.79 ± 0.02	
**Total Phenols**	**N.d.**	**N.d.**	**0.79**	
**Terpenes**				
α-farnesene	0.06 ± 0.01	N.d.	N.d.	
**Total Terpenes**	**0.06**	**N.d.**	**N.d.**	
**Molecule**	**VG4 MATS**	**VG4 PLANK**	**VG4 BIOFILM-DETACHED**	**Odor and Flavor**
**Esters**				
3-methylbutyl acetate	1.25 ± 0.02	0.60 ± 0.01	N.d.	Sweet, fruity, banana
Ethyl acetate	0.54 ± 0.02	0.69 ± 0.02	N.d.	Fruity, sweet, weedy, green, with a grape and cherry nuance
Ethyl decanoate	3.34 ± 0.07	1.69 ± 0.03	N.d.	Sweet, waxy, fruity, apple, grape, oily, brandy
Ethyl dodecanoate	0.15 ± 0.02	N.d.	N.d.	Sweet, waxy, floral, soapy, clean, creamy, dairy, fruity
Ethyl octanoate	0.20 ± 0.01	0.10 ± 0.01	N.d.	Fruity, sweet, apricot, banana, pear, musty, pineapple, dairy, creamy
Nerolidyl acetate	0.06 ± 0.01	N.d.	N.d.	Fresh, sweet, citrus, freesia, woody
Pentyl acetate	N.d.	8.22 ± 0.09	N.d.	Fruity, banana, pear, apple, sweet
Phenethyl pivalate	0.03 ± 0.01	N.d.	0.37 ± 0.01	Rose, spicy, geranium, blueberry, balsamic, grassy
2-phenylethyl acetate	3.69 ± 0.06	3.15 ± 0.04	6.24 ± 0.06	Floral, rose, sweet, honey, fruity, tropical, green
**Total Esters**	**9.29**	**14.46**	**6.62**	
**Alcohols**				
(2R)-2-aminopropan-1-ol	0.01 ± 0.01	N.d.	N.d.	
(Z,E)-farnesol	N.d.	N.d.	0.18 ± 0.02	Delicate floral
2-methylbutan-1-ol	1.47 ± 0.04	0.74 ± 0.02	N.d.	Roasted, winey, fruity, alcoholic
2-phenylethanol	0.28 ± 0.01	0.27 ± 0.01	6.12 ± 0.09	Floral, rose, sweet, bready
3-methylbutan-1-ol	1.38 ± 0.03	0.70 ± 0.01	N.d.	Alcoholic, pungent, cognac, fruity, banana, molasses, fermented,
Pentan-1-ol	7.28 ± 0.09	3.69 ± 0.06	N.d.	Fusel, oily, sweet, balsamic, pungent, fermented, bready, cereal, fruity
Trans-farnesol	N.d.	0.02 ± 0.01	N.d.	Muguet, floral, sweet, lily
**Total Alcohols**	**10.43**	**5.43**	**6.30**	

**Ketons**				
Methyl heptyl ketone	N.d.	N.d.	0.19 ± 0.02	Fruity, sweet, cheesy, herbal, coconut, fresh, green, dairy, buttery
8-hydroxyoctan-2-one	N.d.	N.d.	0,92 ± 0.02	
**Total Ketons**	**N.d.**	**N.d.**	**1,11**	
**Organic acids**				
N-decanoic acid	0,04 ± 0.01	0.01 ± 0.01	N.d.	Rancid, sour, fatty, citrus, creamy, buttery, fruity, dairy
**Total Organic acids**	**0.04**	**0.01**	**N.d.**	
**Phenols**				
2,5-ditert-butylphenol	N.d.	N.d.	0.97 ± 0.02	
**Total Phenols**	**N.d.**	**N.d.**	**0.97**	
**Terpenes**				
α-springene	0,06 ± 0.01	N.d.	N.d.	
**Total Terpenes**	**0.06**	**N.d.**	**N.d.**	
**Molecule**	**CBS834^T MATS**	**CBS834^T PLANK**	**CBS834^T BIOFILM-DETACHED**	**Odor and Flavor**
**Esters**				
2-methylbutyl acetate	0.26 ± 0.01	N.d.	N.d.	Sweet, fruity, banana, tropical, juicy
3-methylbutyl acetate	2.85 ± 0.05	N.d.	N.d.	Sweet, fruity, banana
Ethyl acetate	3.88 ± 0.06	4.50 ± 0.09	N.d.	Fruity, sweet, weedy, green, with a grape and cherry nuance
Ethyl decanoate	N.d.	4.70 ± 0.08	N.d.	Sweet, waxy, fruity, apple, grape, oily, brandy
Ethyl octanoate	N.d.	0.28 ± 0.04	N.d.	Fruity, sweet, apricot, banana, pear, musty, pineapple, dairy, creamy
Ethyl propionate	0.08 ± 0.02		N.d.	Sweet, fruity, juicy, grape, pineapple, fermented, winey, bubble gum, apple
Isopentyl acetate	N.d.	2.45 ± 0.06	N.d.	Sweet, fruity, banana, solvent, ripe, estery, green
Phenethyl isobutyrate	N.d.	N.d.	2.25 ± 0.06	Floral, fruity, rose, tea, peach, yeasty, balsamic, honey
Phenethyl pivalate	N.d.	0.04 ± 0.01	0.19 ± 0.01	Rose, spicy, geranium, blueberry, balsamic, grassy
2-phenylethyl acetate	3.88 ± 0.09	1.91 ± 0.05	0.16 ± 0.03	Floral, rose, sweet, honey, fruity, tropical, green
**Total Esters**	**10.97**	**14.06**	**2.62**	
**Alcohols**				
2-methylbutan-1-ol	3.55 ± 0.02	2.07 ± 0.06	N.d.	Roasted, winey, fruity, alcoholic
2-phenylethanol	1.48 ± 0.01	0.78 ± 0.04	11.45 ± 0.09	Floral, rose, sweet, bready
3-methylbutan-1-ol	3.32 ± 0.03	1.94 ± 0.06	N.d.	Alcoholic, pungent, cognac, fruity, banana, molasses, fermented
4-aminopentan-1-ol	N.d.	0.05 ± 0.01	N.d.	
Isobutanol	N.d.	0.73 ± 0.02	N.d.	Ethereal, winey, fusel, whiskey
**Total Alcohols**	**8.36**	**5.58**	**11.45**	
**Ketons**				
Methyl heptyl ketone	N.d.	N.d.	0.10 ±0.01	Fruity, sweet, cheesy, herbal, coconut, fresh, green, dairy, buttery
8-hydroxyoctan-2-one	N.d.	N.d.	0.55 ± 0.02	
**Total Ketons**	**N.d.**	**N.d.**	**0.66**	
**Organic acids**				
N-decanoic acid	N.d.	0,08 ± 0.01	N.d.	Rancid, sour, fatty, citrus, creamy, buttery, fruity, dairy
**Total Organic acids**	**N.d.**	**0,08**	**N.d.**	
**Phenols**				
2,5-ditert-butylphenol	N.d.	N.d.	0.44 ± 0.02	
**Total Phenols**	**N.d.**	**N.d.**	**0.44**	
	**FM09 MATS**	**FM09 PLANK**	**FM09 BIOFILM-DETACHED**	**Odor and Flavor**
**Esters**				
2-methylbutyl acetate	0.06 ± 0.02		N.d.	Sweet, fruity, banana, tropical, juicy
3-methylbutyl acetate	0.67 ± 0.01	0.58 ± 0.02	N.d.	Sweet, fruity, banana
Ethyl acetate	0.17 ± 0.01	0.92 ± 0.03	N.d.	Fruity, sweet, weedy, green, with a grape and cherry nuance

Ethyl decanoate	N.d.	1.66 ± 0.05	N.d.	Sweet, waxy, fruity, apple, grape, oily, brandy
Ethyl octanoate	N.d.	0.10 ± 0.01	N.d.	Fruity, sweet, apricot, banana, pear, musty, pineapple, dairy, creamy
Pentyl acetate	9.27 ± 0.05	8.05 ± 0.06	N.d.	Fruity, banana, pear, apple, sweet
Pentyl propionate	0.09 ± 0.02	N.d.	N.d.	Sweet, fruity, apricot, pineapple, apple, banana
Phenethyl propionate	0.01 ± 0.01	0.01 ± 0.01	0.06 ± 0.01	Floral, rose, fruity, honey, balsamic, green, sweet, tropical
2-phenylethyl acetate	3.55 ± 0.03	3.08 ± 0.03	0.45 ± 0.02	Floral, rose, sweet, honey, fruity, tropical, green
**Total Esters**	**13.85**	**14.42**	**0.52**	
**Alcohols**				
(2R)-2-aminopropan-1-ol	0.02 ± 0.01	N.d.	N.d.	
(Z,E)-farnesol	N.d.	0.02 ± 0.01	N.d.	Delicate floral
2-methylbutan-1-ol	0.84 ± 0.02	0.73 ± 0.02	N.d.	Roasted, winey, fruity, alcoholic
2-phenylethanol	0.24 ± 0.01	0.17 ± 0.03	7.36 ± 0.09	Floral, rose, sweet, bready
3-methylbutan-1-ol	0.78 ± 0.02	0.68 ± 0.01	N.d.	Alcoholic, pungent, cognac, fruity, banana, molasses, fermented
4-aminopentan-1-ol	0.02 ± 0.01		N.d.	
Pentan-1-ol	4.16 ± 0.03	3.61 ± 0.06	N.d.	Fusel, oily, sweet, balsamic, pungent, fermented, bready, cereal, fruity
Trans-farnesol	0.01 ± 0.01	N.d.	N.d.	Muguet, floral, sweet, lily
**Total Alcohols**	**6.09**	**5.21**	**7.36**	
**Ketons**				
Methyl heptyl ketone	N.d.	N.d.	0.19 ± 0.02	Fruity, sweet, cheesy, herbal, coconut, fresh, green, dairy, buttery
8-hydroxyoctan-2-one	N.d.	N.d.	1.15 ± 0.02	
**Total Ketons**	**N.d.**	**N.d.**	**1.35**	
**Organic acids**				
N-decanoic acid	N.d.	0.02 ± 0.01	N.d.	Rancid, sour, fatty, citrus, creamy, buttery, fruity, dairy
**Total Organic acids**	**N.d.**	**0.02**	**N.d.**	
**Phenols**				
2,5-ditert-butylphenol	N.d.	N.d.	0.33 ± 0.01	
**Total Phenols**	**N.d.**	**N.d.**	**0.33**	
**Molecule**	**1SC4 MATS**	**1SC4 PLANK**	**1SC4 BIOFILM-DETACHED**	**Odor and Flavor**
**Esters**				
2-methylbutyl acetate	0.14 ± 0.01	0.09 ± 0.01	N.d.	Sweet, fruity, banana, tropical, juicy
3-methylbutyl acetate	1.34 ± 0.02	1.07 ± 0.02	N.d.	Sweet, fruity, banana
3-methylbutyl propionate	N.d.	0.07 ± 0.03	N.d.	Sweet, pineapple, tropical fruit, apple, banana, fresh green, melon
Ethyl 9-decenoate	N.d.	0.01 ± 0.01	N.d.	Fruity fatty
Ethyl acetate	1.31 ± 0.03	2.00 ± 0.03	N.d.	Fruity, sweet, weedy, green with a grape and cherry nuance
Ethyl decanoate	4.45 ± 0.05	3.03 ± 0.05	N.d.	Sweet, waxy, fruity, apple, grape, oily, brandy
Ethyl octanoate	0.27 ± 0.05	0.18 ± 0.02	N.d.	Fruity, sweet, apricot, banana, pear, musty, pineapple, dairy, creamy
Phenethyl pivalate	N.d.	N.d.	0.63 ± 0.02	Rose, spicy, geranium, blueberry, balsamic, grassy
Phenethyl propionate	0.01 ± 0.01	0.01 ± 0.01	N.d.	Floral, rose, fruity, honey, balsamic, green, sweet, tropical
2-phenylethyl acetate	7.92 ± 0.03	10.59 ± 0.09	0.41 ± 0.01	Floral, rose, sweet, honey, fruity, tropical, green
**Total Esters**	**15.46**	**17.09**	**1.05**	
**Alcohols**				
Trans-farnesol		0.01 ± 0.01	N.d.	Muguet, floral, sweet, lily
2-methylbutan-1-ol	1.96 ± 0.03	1.33 ± 0.03	N.d.	Roasted, winey, fruity, alcoholic
2-phenylethanol	0.51 ± 0.01	0.17 ± 0.02	7.40 ± 0.09	Floral, rose, sweet, bready
3-methylbutan-1-ol	1.83 ± 0.03	1.25 ± 0.06	N.d.	Alcoholic, pungent, cognac, fruity, banana, molasses, fermented
4-aminopentan-1-ol	0.08 ± 0.01	N.d.	N.d.	
**Total Alcohols**	**4.38**	**2.78**	**7.40**	
**Ketons**				

Methyl heptyl ketone	N.d.	N.d.	0.32 ± 0.01	Fruity, sweet, cheesy, herbal, coconut, fresh, green, dairy, buttery
8-hydroxyoctan-2-one	N.d.	N.d.	0.01 ± 0.01	
**Total Ketons**	**N.d.**	**N.d.**	**0.33**	
**Organic acids**				
Acetic acid	N.d.	0.07 ± 0.01	N.d.	Acidic, sharp, pungent, sour, vinegar, fruit overripe, fruit acetic
**Total Organic acids**	**N.d.**	**0.07** ± 0.02	**N.d.**	
**Phenols**				
2,5-ditert-butylphenol	N.d.	N.d.	1.17 ± 0.03	
**Total Phenols**	**N.d.**	**N.d.**	**1.17**	
**Terpenes**				
α-springene	0.03 ± 0.01	N.d.	N.d.	
**Total Terpenes**	**0.03**	**N.d.**	**N.d.**	

Molecule	LM142 MATS	LM142 PLANK	LM142 BIOFILM-DETACHED	Odor and Flavor
**Esters**				
2-methylbutyl acetate	N.d.	0.05 ± 0.01	N.d.	Sweet, fruity, banana, tropical, juicy
2-phenethyl butanoate	N.d.	N.d.	7.25 ± 0.09	
3-methylbutyl acetate	0.67 ± 0.01	0.64 ± 0.02	N.d.	Sweet, fruity, banana
Ethyl acetate	0.29 ± 0.01	0.88 ± 0.02	N.d.	Fruity, sweet, weedy, green, with a grape and cherry nuance
Ethyl decanoate	N.d.	1.81 ± 0.03	N.d.	Sweet, waxy, fruity, apple, grape, oily, brandy
Ethyl octanoate	0.11 ± 0.01	0.11 ± 0.01	N.d.	Fruity, sweet, apricot, banana, pear, musty, pineapple, dairy, creamy
Nerolidyl acetate	0.01 ± 0.01	N.d.	N.d.	Fresh, sweet, citrus, freesia, woody
Pentyl acetate	9.22 ± 0.06	8.81 ± 0.09	N.d.	Fruity, banana, pear, apple, sweet
Phenethyl hexanoate	0.13 ± 0.01	N.d.	N.d.	Sweet, honey, floral, woody, green, banana, pineapple
Phenethyl pivalate	N.d.	0.02 ± 0.01	0.33 ± 0.01	Rose, spicy, geranium, blueberry, balsamic, grassy
Phenethyl propionate	0.01 ± 0.01	N.d.	N.d.	Floral, rose, fruity, honey, balsamic, green, sweet, tropical
2-phenylethyl acetate	3.53 ± 0.06	2.00 ± 0.03	0.34 ± 0.03	Floral, rose, sweet, honey, fruity, tropical, green
**Total Esters**	**14.00**	**14.35**	**7.93**	
**Alcohols**				
2-methylbutan-1-ol	0.83 ± 0.02	0.80 ± 0.02	N.d.	Roasted, winey, fruity, alcoholic
2-phenylethanol	0.19 ± 0.01	0.18 ± 0.01	3.98 ± 0.06	Floral, rose, sweet, bready
3-(2-methyl-1,3-dioxan-2-yl)propan-1-ol	N.d.	N.d.	0.17 ± 0.02	
3-methylbutan-1-ol	0.67 ± 0.02	0.64 ± 0.02	N.d.	Alcoholic, pungent, cognac, fruity, banana, molasses, fermented
4-aminopentan-1-ol	0.01 ± 0.01	N.d.	N.d.	
4-butoxybutan-1-ol	N.d.	N.d.	0.11 ± 0.01	
8-methylnonane-1,8-diol	N.d.	N.d.	0.32 ± 0.01	
Pentan-1-ol	4.14 ± 0.06	3.95 ± 0.03	N.d.	Fusel, oily, sweet, balsamic, pungent, fermented, bready, cereal, fruity
Piperidin-3-ol	N.d.	0.02 ± 0.01	N.d.	
Trans-farnesol	0.01 ± 0.01	N.d.	N.d.	Muguet, floral, sweet, lily
**Total Alcohols**	**5.88**	**5.60**	**4.60**	
**Ketons**				
Methyl heptyl ketone	N.d.	N.d.	0.17 ± 0.01	Fruity, sweet, cheesy, herbal, coconut, fresh, green, dairy, buttery
8-hydroxyoctan-2-one	N.d.	N.d.	0.33 ± 0.01	
**Total Ketons**	**N.d.**	**N.d.**	**0.51**	
**Phenols**				
2,5-ditert-butylphenol	N.d.	N.d.	0.65 ± 0.02	
**Total Phenols**	**N.d.**	**N.d.**	**0.65**	
**Terpenes**				
β-springene	0.02 ± 0.01	N.d.	0.55 ± 0.01	

Total Terpenes	0.02	N.d.	0.55 ±	
**Molecule**	**M83 MATS**	**M83 PLANK**	**M83 BIOFILM-DETACHED**	**Odor and Flavor**
**Esters**				
Ethyl acetate	1.71 ± 0.01	0.67 ± 0.01	N.d.	Fruity, sweet, weedy, green, with a grape and cherry nuance
2-phenylethyl acetate	9.76 ± 0.03	3.64 ± 0.06	5.46 ± 0.06	Floral, rose, sweet, honey, fruity, tropical, green
3-methylbutyl acetate	0.27 ± 0.01	0.11 ± 0.01	N.d.	Sweet, fruity, banana
Pentyl acetate	3.57 ± 0.06	9.49 ± 0.06	N.d.	Fruity, banana, pear, apple, sweet
Ethyl pyruvate	0.10 ± 0.01	N.d.	N.d.	Fruity, sweet, sharp, vegetable, caramelly, ethereal
Ethyl decanoate	0.73 ± 0.03	1.95 ± 0.01	N.d.	Sweet, waxy, fruity, apple, grape, oily, brandy
Ethyl octanoate	0.04 ± 0.01	N.d.	N.d.	Fruity, sweet, apricot, banana, pear, musty, pineapple, dairy, creamy, fatty
Nerolidyl acetate	0.08 ± 0.01	N.d.	N.d.	Fresh, sweet, citrus, freesia, woody
Isopropyl hexadecanoate	0.11 ± 0.01	N.d.	N.d.	Oily
Phenethyl pivalate	N.d.	0.02 ± 0.01	0.42 ± 0.01	Rose, spicy, geranium, blueberry, balsamic, grassy
**Total Esters**	**16.41**	**15.90**	**5.88**	
**Alcohols**				
(2R)-2-aminopropan-1-ol	0.06 ± 0.01	0.02 ± 0.01	N.d.	
3-methylbutan-1-ol	0.26 ± 0.02	0.69 ± 0.02	N.d.	Alcoholic, pungent, cognac, fruity, banana, molasses, fermented
2-methylbutan-1-ol	0.32 ± 0.03	0.30 ± 0.01	N.d.	Roasted, winey, fruity, alcoholic
Pentan-1-ol	1.60 ± 0.01	4.26 ± 0.03	N.d.	Fusel, oily, sweet, balsamic, pungent, fermented, bready, cereal, fruity
2-phenylethanol	0.50 ± 0.01	0.14 ± 0.01	7.70 ± 0.09	Floral, rose, sweet, bready
1-aminopropan-2-ol	N.d.	0.03 ± 0.01	N.d.	Fishy, ammonia like
4-aminopentan-1-ol	N.d.	0.02 ± 0.01	N.d.	
4-butoxybutan-1-ol	N.d.	N.d.	0.18 ± 0.01	
**Total Alcohols**	**2.76**	**5.48**	**7.88**	
**Ketons**				
Methyl heptyl ketone	N.d.	N.d.	0.22 ± 0.01	Fruity, sweet, cheesy, herbal, coconut, fresh, green, dairy, buttery
8-hydroxyoctan-2-one	N.d.	N.d.	0.38 ± 0.02	
**Total Ketons**	**N.d.**	**N.d.**	**0.60**	
**Lactones**				
D-galactonic acid, γ-lactone	0.04 ± 0.01	N.d.	N.d.	
**Total Lactones**	**0.04**	**N.d.**	**N.d.**	
**Organic acids**				
Acetic acid	N.d.	0.12 ± 0.01	N.d.	Acidic, sharp, pungent, sour, vinegar, fruit overripe, fruit acetic
N-decanoic acid	N.d.	0.02 ± 0.01	N.d.	Rancid, sour, fatty, citrus, creamy, buttery, fruity, dairy
**TOT Organic acids**	**N.d.**	**0.14**	**N.d.**	
**Phenols**				
2,5-ditert-butylphenol	N.d.	N.d.	0.36 ± 0.01	
**Total Phenols**	**N.d.**	**N.d.**	**0.36**	
